# Emergent dispersal networks in dynamic wetlandscapes

**DOI:** 10.1038/s41598-020-71739-8

**Published:** 2020-09-07

**Authors:** Leonardo E. Bertassello, Antoine F. Aubeneau, Gianluca Botter, James W. Jawitz, P. S. C. Rao

**Affiliations:** 1 Lyles School of Civil Engineering, Purdue University, West Lafayette, IN 47907-2051 USA; 2 Department of Civil, Architectural and Environmental Engineering, University of Padua, 35100 Padua, Italy; 3 Soil and Water Sciences Department, University of Florida, Gainesville, FL 32611 USA; 4 Agronomy Department, Purdue University, West Lafayette, IN 47907-2054 USA

**Keywords:** Ecological networks, Hydrology, Geomorphology

## Abstract

The connectivity among distributed wetlands is critical for aquatic habitat integrity and to maintain metapopulation biodiversity. Here, we investigated the spatiotemporal fluctuations of wetlandscape connectivity driven by stochastic hydroclimatic forcing, conceptualizing wetlands as dynamic habitat nodes in dispersal networks. We hypothesized that spatiotemporal hydrologic variability influences the heterogeneity in wetland attributes (e.g., size and shape distributions) and wetland spatial organization (e.g., gap distances), in turn altering the variance of the dispersal network topology and the patterns of ecological connectivity. We tested our hypotheses by employing a DEM-based, depth-censoring approach to assess the eco-hydrological dynamics in a synthetically generated landscape and three representative wetlandscapes in the United States. Network topology was examined for two end-member connectivity measures: centroid-to-centroid (C2C), and perimeter-to-perimeter (P2P), representing the full range of within-patch habitat preferences. Exponentially tempered Pareto node-degree distributions well described the observed structural connectivity of both types of networks. High wetland clustering and attribute heterogeneity exacerbated the differences between C2C and P2P networks, with Pareto node-degree distributions emerging only for a limited range of P2P configuration. Wetlandscape network topology and dispersal strategies condition species survival and biodiversity.

## Introduction

Dispersal of species among patchy habitats is important in many ecosystems, including marine, freshwater and terrestrial environments^1–3^. Heterogeneous habitats result from evolution of the landscape (e.g., topography) under vegetation (e.g., land cover) and climate controls^4,5^. Heterogeneity and connectivity are key features of patch-habitat complexity, which in turn constrains habitat suitability and accessibility. Habitat heterogeneity is apparent in the diversity of patch sizes and shapes and their abundance. Patch connectivity, the degree to which landscape affects dispersal of organisms, is affected by the spatial organization (e.g., gap distances). Patch connectivity is a primary control of many ecological processes^6^, including population movement^7^, changes in species diversity^8^, and metacommunity dynamics^9^. Losses of patches and connectivity are thus a central concern in conservation biology^10,11^, but the diversity of niche-habitats is also important for the biodiversity in local patches and across the landscape^12^.

We focus here on wetland-rich landscapes (henceforth, *wetlandscapes*) as an ecologically representative case study for aquatic patchy habitats. Wetlands, and the unique biota they host, are among the most threatened ecosystems in the world^13^ because of climate change and other anthropogenic factors such as land use change. Wetlands occur as discrete, fragmented patches within a matrix of heterogeneous upland habitats^14^, and wetland-dependent metapopulations exchange individuals dispersing through the uplands^15,16^. Spatiotemporal variations in wetland habitat complexity, driven by external forcing, affect habitat suitability, and thus the persistence of biota within and across wetlands^17–19^. Here, a single wetland is seen as a patch that can change in size and shape, and eventually disappear, in response to changes in hydroclimatic conditions. This, in turn, modulates the species dispersal among individual wetlands and connectivity across the landscape.

In wetlandscapes, topography constrains the abundance and spatial organization of patches^20,21^, i.e., depressions filled with water and suitable as habitats. Landscape surface roughness, quantified using the Hurst exponent, $0<H<1$, ^22,23^ influences how wetlands fill and merge with changes in water levels. $H$ represents the scale of spatial correlation between the elevation points of a topographic surface. When $H\to 1.0$, the correlation among the elevation points is large, and surfaces are relatively smooth, whereas for $H\to 0.0$, the correlation decreases for rougher surfaces^24^. High correlation among terrain elevations (large $H$) also suggests a more regular pattern in the spatial distribution of wetland patches rather than random distributions. Landscape complexity and variations in hydrological conditions affect the structure and dynamics of depressional (wetland) patterns, and thus the dispersal network that connects them. In wetlands where the hydrology is driven directly by rainfall, water inputs are censored by exfiltration and evapotranspiration^25^. In wetlands connected to shallow groundwater, the indirect effects of the hydroclimatic forcing are reflected in synchronous temporal fluctuations in groundwater and wetland stages^26^. Such stochasticity in hydroclimatic forcing contributes to the spatiotemporal fluctuations in heterogeneity and connectivity of patch habitats.

In this paper, we address the following overarching research question: how does temporal variability in hydrological conditions affect ecological connectivity of wetland-patch habitats? We posit that the variability in wetland attributes (e.g., size, shape, bathymetry) and spatial organization (e.g., inter-patch gap distances) should condition the patch-network topology. Our guiding hypothesis is that heterogeneous patch habitats will yield complex dispersal networks (e.g. scale free networks), while homogeneous and sparse habitats will foster simpler dispersal network topologies (e.g., random networks).

**Figure 1:**
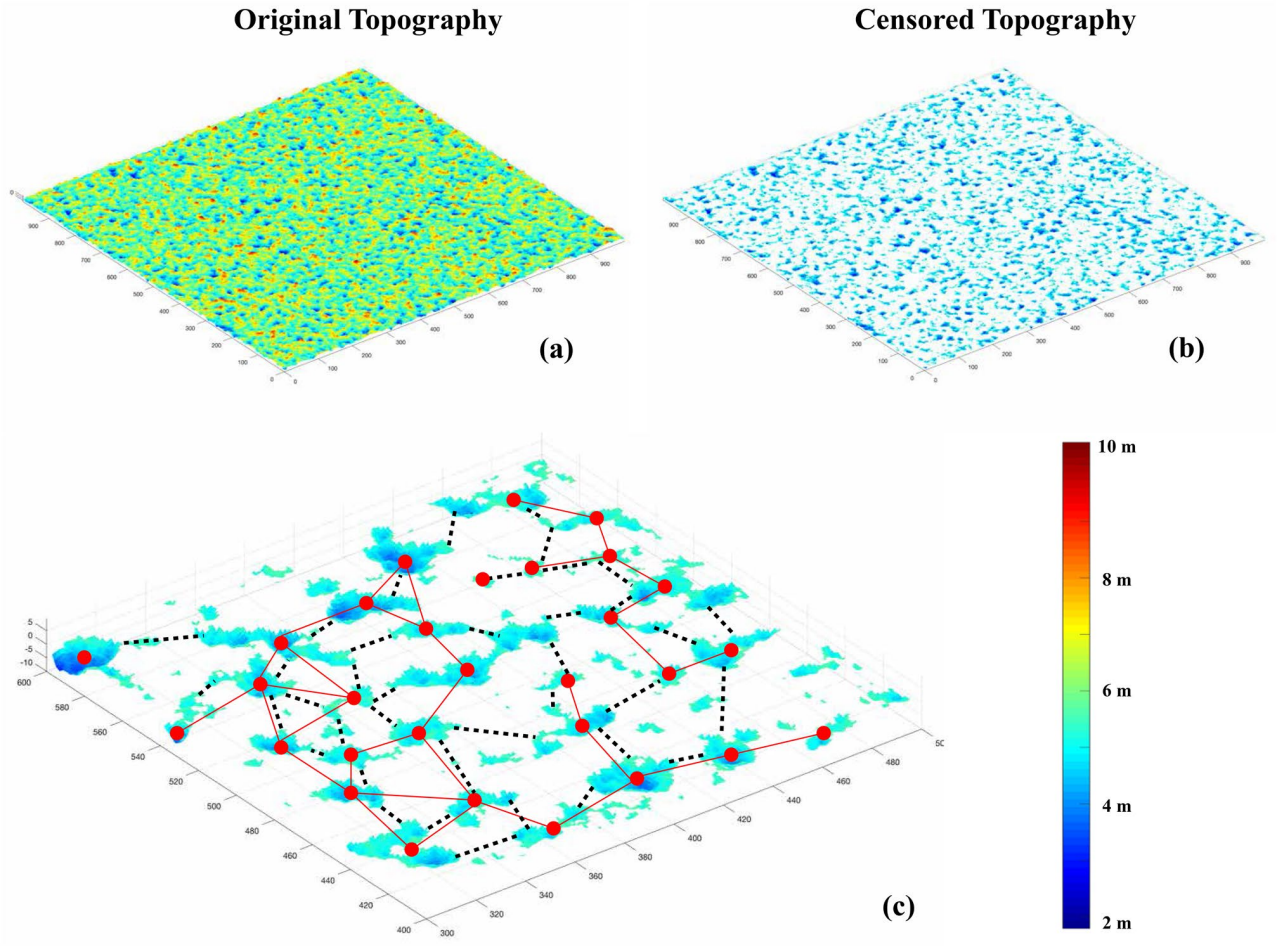
Graphical representation of the landscape hydrological model used to identify wetlands using DEMs. The process involves slicing the original DEM (**a**) using a series of 2D planes that follow the mean topography of the spatial domain (see “Methods”). The intersection between the 2D plane and the original topography identifies all depressions below the 2D plane as wetlands (**b**). Differences in water levels resulting from wetland bathymetry are shown in blue shades. For each sliced topographic surface, we computed the emergent wetlandscape network by connecting a given pair of wetlands only if the gap distance (either C2C and P2P) was less or equal to a given threshold distance, $D$.

We used a DEM-based, depth-censoring approach to emulate changes in wetland hydrological attributes (e.g., wetted surface area; wetted perimeter) during the wetland filling-merging process (Fig. 1). A similar level-set method was recently proposed^27^ for analyses of large-scale DEM data for characterizing nested, hierarchical depressions in wetlandscapes. Our approach focuses on factors that dominate the emergence of landscape-scale patterns despite local-scale heterogeneities. We analyzed DEM for four case studies: a synthetically generated fractal landscape as an idealized fractal habitat, and three U.S. wetlandscapes representing diverse wetlandscapes (see “Methods”). The spatial complexity of these wetlandscapes was measured by their heterogeneity (area and perimeter distributions) and spatial organization (gap distances).

First, we examined how surface roughness (Hurst Coefficient) influences the wetland filling process, and the associated changes in wetland abundance and attributes. Second, we assessed how spatial heterogeneity in wetland attributes dynamically influences patch connectivity at the landscape scale (30 × 30 km domain). We compared the topology of wetland dispersal networks by connecting patches (nodes) using two criteria: (1) *perimeter-to-perimeter distance*, P2P^28^, and (2) *centroid-to-centroid distance*, C2C^29–31^. Network connectivity based on these two criteria represents the complete range of preferred habitat niches within habitat patches. Comparing C2C and P2P networks allows for the identification of the differences in conceptualizing wetlands merely as nodes (C2C networks, with centroids as points in 2-D space), or wetlands as 3-D objects, defined by bathymetry^32^. Based on C2C and P2P criteria for patch-connectivity, we compared the dynamic topology of wetland networks over the range of hydrologic conditions (full to dry). We close with a discussion of implications of network topological dynamics in patchy habitats for species dispersal.

## Methods

### Wetlandscape hydrological model

Following Bertassello et al.^26^, we simulated the spatial hydrologic dynamics in wetlandscapes by assuming that landscape topography and shallow groundwater regulate the number of inundated wetlands and their attributes. Here, all portions of the landscape located at elevations lower than the censoring level are assumed to be filled with water. This generalized model for wetland hydrology allows for the estimation of wetland attributes in terms of stage, wetted-surface area, storage volume and wetted perimeter. Among these attributes, here we are focus on the number of inundated wetlands and their total wetted perimeter because they are fundamental to describe the density and the shape of the patchy habitats scattered in wetlandscapes. A higher density of wetlands provides more solutions for the dispersal of semi-aquatic species, as well as larger number of wetlands offer habitat diversity and higher carrying capacity. Wetland perimeter is considered here an index for habitat availability for semi-aquatic species, such amphibians associated with these transitional wet and dry habitats surrounding wetlands^46^.

The main assumption of our approach is considering the sequence of censoring depths as parallel to the mean slope of the terrain. When the slope of the terrain is equal to zero, the censoring level results in a horizontal xy-planes that intersect the landscape topography at different levels^47^. In the general case, however, the slope of the terrain is not zero, an instance that makes the description of the problem more complicated. However, by applying a change of coordinates, and thus detrending the landscape topography^26^, we can simplify the problem and refer to a unique censoring level, ${z}_{gw}$, across the entire domain. In this way, censoring the original topography using a censoring level that follows the mean slope of the terrain is equivalent to censoring a detrended DEM using a horizontal 2D-plane located in correspondence of the mean elevation of the landscape.

### Case studies and data

Our framework requires only a DEM as an input to identify the spatial patterns and size/shape distributions of all potential wetlands. The DEM data used in our analysis were obtained from the Unites States Geological Survey (USGS) National Map Viewer^48^, and are reported as background in Figure S1. The DEM data are 1/3 arc-second resolution (10 × 10 m), and a vertical accuracy of 1 m. We tested our framework in three 30 × 30 km wetlandscapes across the United States. The three wetlandscapes are characterized by different wetland densities and wetted areas (Figure S1). The wetlandscape in Texas has the smallest density (~ 1 wetland/km^2^), compared with Florida (~ 15 wetlands/km^2^) and N. Dakota (~ 29 wetlands/km^2^). Instead, considering wetland coverage, Florida is the wetlandscape characterized by the largest value (~ 50%), then N. Dakota (~ 13%) and Texas (~ 4%). These considerations have important implications for the distribution of separation distances between wetlands.

We also assessed the spatial network dynamics in a synthetic generated landscape. We used the Inverse Fourier Transform (IFT) method to generate fractal surfaces following Gallant et al.^49^. The fractal surface is a superposition of waves of normally distributed random amplitude (e.g. standard deviation $$\sigma =2$$ m) with random phase shifts uniformly distributed in the interval $$\left[\mathrm{0,2}\pi \right]$$. The power spectrum, $$G(f)$$^50^, of a fractal surface is an inverse power-law function of frequency, $$f$$ [L^-1^], $$G\left(f\right) \sim {f}^{-\beta }$$, where the exponent is related to the Hurst coefficient, $$H$$, as $$\beta =\left(1+2H\right)$$$$\in [\mathrm{1,3}]$$. The surface is produced by taking the Inverse Fourier Transform of the synthetically generated spectrum, $$G(f)$$, using the fast Fourier transform (FFT) algorithm^49–51^. With two parameters (H and $$\sigma$$) we are able to create landscapes that resemble the characteristics of actual topographic surfaces.

These synthetic surfaces represent idealized landscapes where our conceptual framework was evaluated as a reference case. For example, the application of the generalized hydrologic model does not need to account for landscape detrending since the slope of the synthetic terrain is zero. In what follows, we show the results of the application of our framework to a synthetic landscape generated using H = 0.65 and $\sigma =2$ m., and three wetlandscapes in the U.S.

### Network analysis

Network theory is a suitable tool to represent a landscape of habitat patches as a set of nodes (wetlands) connected to some extent by links between nodes^30^. A link is established if and only if there is a potential for flux between the nodes^52^. Here, we conceptualize this flux as the potential dispersal of semi-aquatic species (e.g., amphibians) from one wetland to another. Accordingly, two wetlands are linked if the distance between them is less than or equal to the maximum distance defined by the dispersal ability of a given species^31,53^. We investigate wetland connectivity for species dispersal by constructing the network based on either the perimeter-to-perimeter distance, P2P^28^, or the centroid-to-centroid distance, C2C^29–31^. These two approaches (Fig. 1) represent the end members of a spatial gradient of preferred habitat zones within wetlands occupied by diverse species. Variation in the hydroclimatic forcing, and thus in the landscape censoring level, has different effects on the C2C and P2P networks. While the variation in wetland size or perimeter does not affect the C2C network since the location of the centroid remains the same, in P2P networks node geometry variability is explicitly considered. Fluctuations in the censoring level (Fig. 1) can increase wetland size facilitating connections between previously isolated patches due to the reduced P2P separation distance or decrease the connectivity when the censoring threshold is low such that few wetlands are inundated and located far apart.

The structural connectivity of the resulting networks is examined by focusing on the node-degree distribution of wetlandscapes. The node-degree distribution is expressed as the complementary cumulative distribution function (CCDF), $\mathrm{P}\left(X>k\right)$ where *k* represents the node-degree. The numerical node-degree distributions are then fitted using an exponentially tempered Pareto distribution. The advantage of this type of distribution is its flexibility. Indeed, as the constant *c* approaches zero, the distribution is a true power law, and the network itself is scale-free. Instead, when $c>$ 0, we observe a tempering of the distribution, due to hydrological or geometrical properties of the wetland habitat, which tend to behave as random networks^54^.

## Results

### Wetlandscape structural complexity

**Figure 2:**
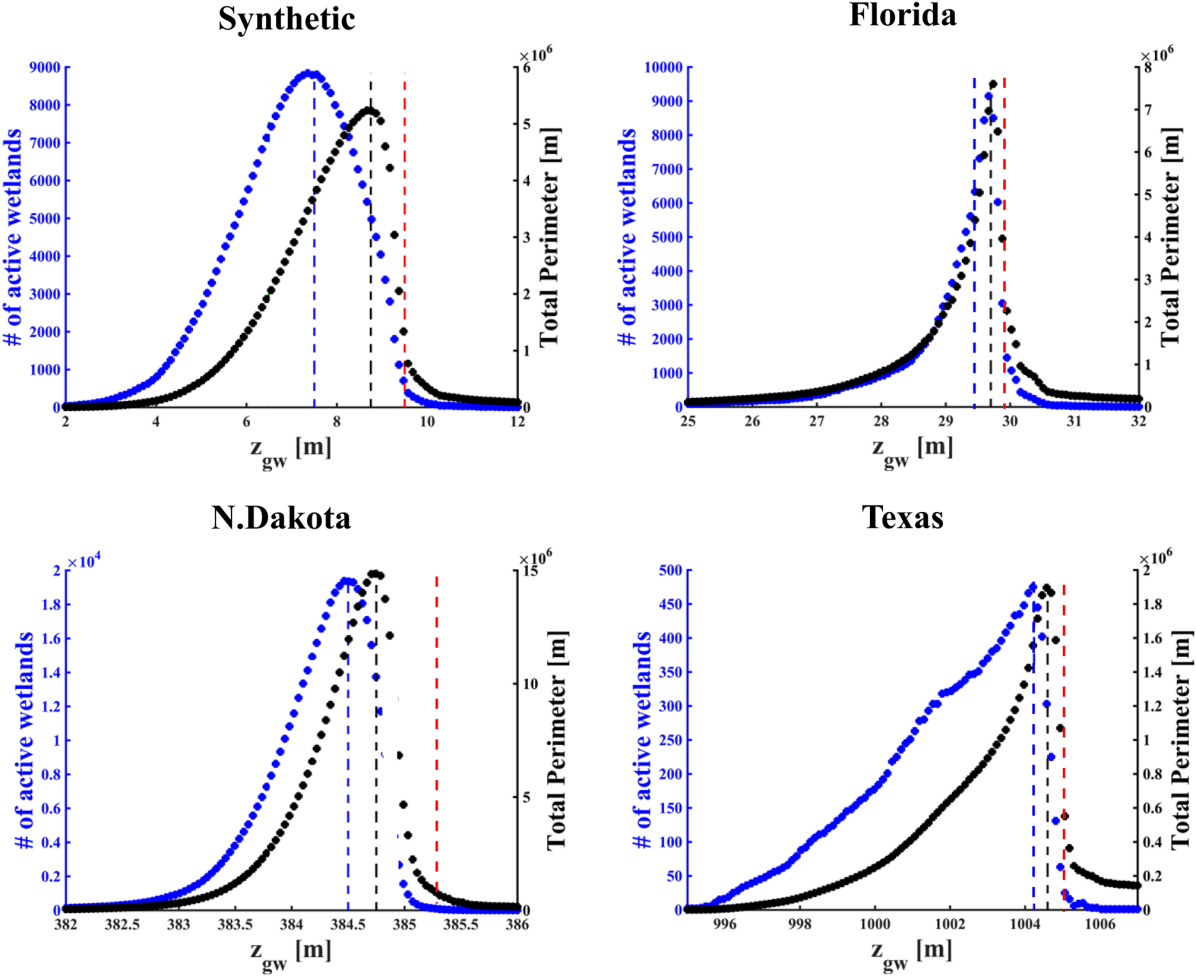
Comparison between the trends for the total number of active wetlands (blue circles) and the total perimeter (black circles) for different censoring levels, ${\mathrm{z}}_{\mathrm{gw}}$, for DEMs of four landscapes. Dashed lines represent the thresholds that were used to compute the emergent ecological network, shown in Fig. 3. The blue and black dashed lines represent the thresholds that maximizes the number of active wetland and the total perimeter, respectively. The red dashed line stands for a censoring level ${\mathrm{z}}_{\mathrm{gw}}>{\mathrm{z}}_{\mathrm{tp}}$.

Decrease in wetland stage (here, censoring the DEM at different depth thresholds, ${z}_{gw}$) is reflected in reduction of both the wetland abundance and their total wetted perimeter. For all four case studies, and for both metrics, an asymmetric (left-skewed), unimodal shape (Fig. 2) characterizes the dependence on ${z}_{gw};$ sharp peaks suggest that small variations in ${z}_{gw}$ cause a rapid shift in both metrics. Two censoring levels, ${z}_{gw}={z}_{aw}$ and ${z}_{gw}={z}_{tp}$, maximize respectively the number of inundated wetlands and the total wetland perimeter for the synthetic landscape and North Dakota. In two other wetlandscapes, the two peaks are either close together (Texas) or completely superimposed (Florida). With lower groundwater levels under drier conditions (e.g., ${z}_{gw}<{z}_{aw}$), few inundated wetlands are present, with small total area and perimeter; thus, the total available wetland habitat is limited. For wetter conditions (e.g., ${z}_{gw}>{z}_{tp}$), few large wetlands are present in a flooded landscape, and small total perimeter limits the habitat available for species preferring wetland edges. Sharp peaks and nonlinear changes in patch attributes and patch density driven by the dynamic hydrologic regime have direct impacts on the habitat capacity, proportional to total wetted-area or total wetted-perimeter. These changes also have significant implications to wetland connectivity, as discussed in “Case studies and data” section.

Results for all four wetlandscapes (Fig. 2) suggest that the wetland-filling process (rising limb) is gradual with changing ${z}_{gw}$ when compared to the sharp drop during the wetland-merging process (falling limb). Differences among the four case studies are related to the differences in the Hurst exponent, H, as the metric for surface roughness. The Texas wetlandscape is smooth and uniform (H = 0.98 $\pm$ 0.008), thus the filling process driven by the intersection of several censoring levels is almost linear. Surface roughness increases for both the North Dakota wetlandscape (H = 0.79 $\pm$ 0.06) and the synthetic fractal landscape (H = 0.65 $\pm$ 0.003), which contributes to quasi-linear trend in the filling process. Florida wetlandscape exhibits the largest surface roughness (with lower H = 0.57 $\pm$ 0.11), which explains the strong non-linear trend during the wetland filling process, and contributes to an abrupt increase in wetland abundance and total wetted perimeter, even for small increases in the censoring level, ${z}_{gw}$. For larger the Hurst exponents, the filling process is less sensitive to variation in the censoring level, ${z}_{gw}$. This pattern is demonstrated by fitting the rising limb of the number of active wetlands and the total perimeter with a power function of the stage. The exponents of this power-law relationship for the number of active wetlands and the total perimeter are inversely correlated with the Hurst exponent with respectively ${R}^{2}=0.85$ and ${R}^{2}=0.90$ (see Supporting Information). These four landscapes also have different spatial heterogeneity in surface roughness. The H values are essentially uniform for the synthetic landscape and Texas wetlandscape, but have distinct spatial heterogeneity of H for Florida and North Dakota wetlandscapes (see Supporting Information), explaining the differences in patterns of filling and merging.

### Wetlandscape spatial organization

**Figure 3:**
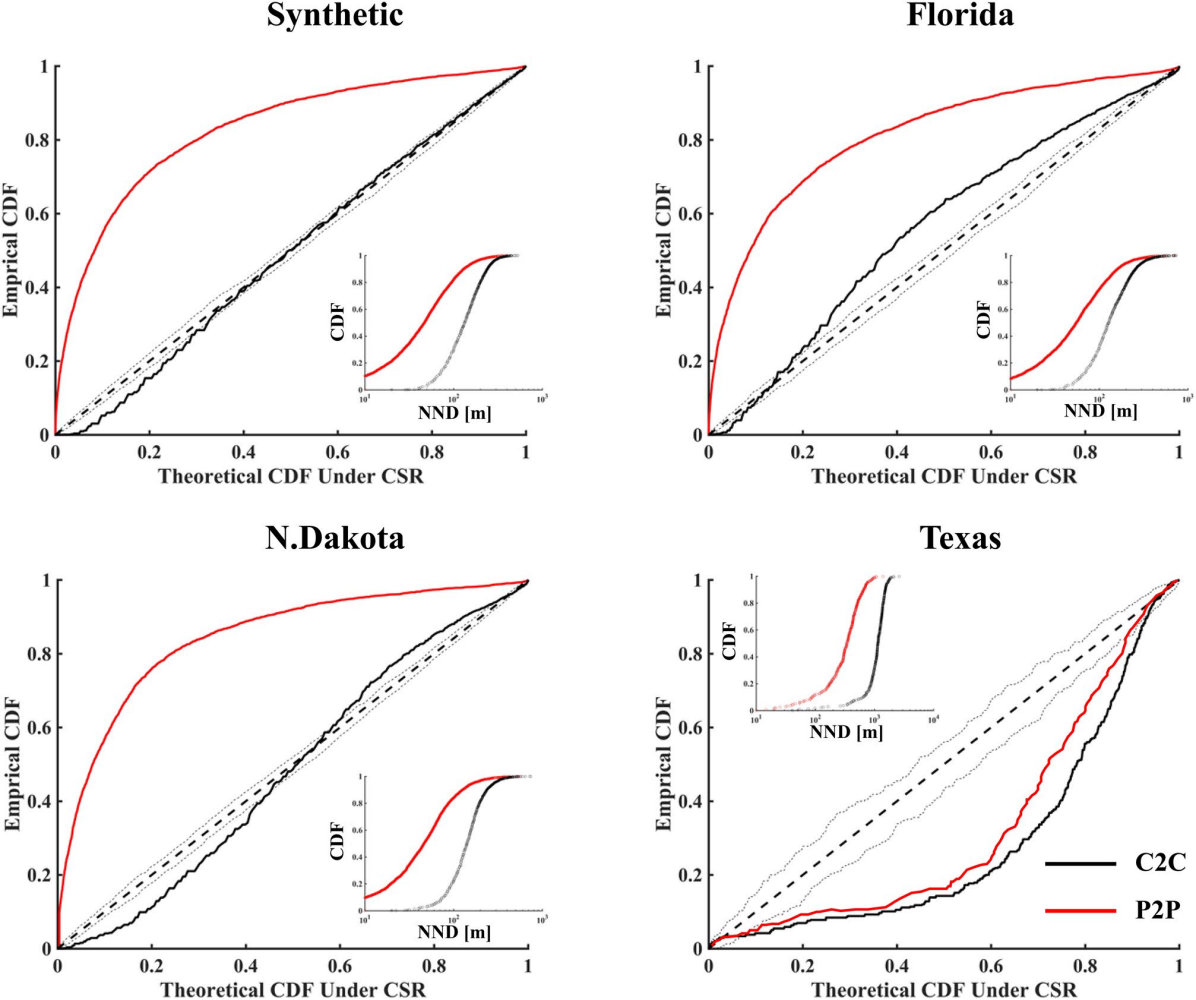
Comparison between the empirical distributions of C2C (black solid line) and P2P (red solid line) against the theoretical Poisson distribution prescribed by a CSR process. When the data follow the 1:1 line, the spatial point pattern is described by a CSR process. Dashed lines show the upper and lower envelopes for 99 simulation of CSR process. Data shown are for comparison between the C2C and P2P gap distances with ${\mathrm{z}}_{\mathrm{gw}}={\mathrm{z}}_{\mathrm{tp}}$. Comparison between the CDF for nearest neighbor separation distance (NND) for both the C2C and P2P distance are shown as insets.

We analyzed the spatial distribution of wetlands by conceptualizing them as realizations of a 2-D point process. In particular, we compared the gap distances for our four case studies (Fig. 3), testing for complete spatial randomness (henceforth, CSR) of both C2C and P2P gap distances. When the hypothesis of CSR is satisfied, a spatial point-process in any planar region $A$ is described by a Poisson distribution^42,43^, with mean $\lambda A$, where $\lambda$ is the average density of points in the spatial domain. Figure 3 shows the comparison between the C2C and P2P nearest neighbor distances obtained when ${z}_{gw}={z}_{tp}$. Compared to the theoretical Poisson distribution, the empirical distributions for the C2C gap distances in Florida, N. Dakota and the synthetic fractal landscape are almost entirely within the envelope of the 1:1 line. In contrast, for these three wetlandscapes the distributions of the P2P gap distances are well above the 1:1 line, indicative of strong clustering^44^. On the other hand, for the simpler geometry of the Texas Playa Lakes (i.e., sparse, nearly circular, and homogeneous wetland areas^21,45^), the C2C and P2P patterns are similar, both distributed below the 1:1 line, suggesting spatial regularity.

Differences between the C2C and P2P gap distances are mainly related to wetland heterogeneity (shape and sizes). For more heterogeneous wetland sizes/shapes, there is greater difference between C2C and P2P gap distances. This difference is evident in the empirical CDFs of gap distances (insets of Fig. 3), with larger heterogeneity in the P2P gap distributions compared to corresponding C2C distributions. This is also quantified by the coefficient of variation (CV), with P2P values approximately double those for C2C for all the analyzed cases (Table 1). While the gap distributions in Florida, N. Dakota, and the fractal landscape are similar, two CDFs obtained for the C2C and P2P distance. Indeed, the shape of the two curves is the same, and they are only translated by their mean that is function of wetland radii. This trend is due to the homogeneity in wetland size and shape that characterize Texas Playa Lakes.

**Table 1:** Summary of the statistics for gap distribution calculated based on the C2C and P2P criteria in the four case studies.

Landscape	C2C			P2P		
	${\mu }_{C2C}$[m]	${\sigma }_{C2C}$[m]	CV_C2C_	${\mu }_{P2P}$[m]	${\sigma }_{P2P}$[m]	CV_P2P_
Synthetic	144	71	0.49	61	55	0.90
N. Dakota	151	73	0.48	60	57	0.95
Florida	148	87	0.59	75	73	0.96
Texas	915	426	0.47	346	319	0.92

Data shown are for comparison between the C2C and P2P gap distances with ${\mathrm{z}}_{\mathrm{gw}}={\mathrm{z}}_{\mathrm{tp}}$.

### Dynamic patch connectivity

Structural connectivity of the wetlandscape networks was evaluated by comparing the empirical node-degree ($k$) distributions for several censoring levels, ${z}_{gw}$. To maintain a constant ratio of threshold distance *D* to mean separation distance in each case, we used $D=200$ m in Florida, N. Dakota and synthetic generated landscape, and $D=1000$ m for Texas; in all cases the ratio $D/{\mu }_{P2P}$ is about 3. The sensitivity of $\mathrm{P}\left(X>k\right)$ to different distance thresholds, $D$, is assessed in Supporting Information for both the C2C and P2P networks. In each case study, the spatiotemporal dynamics of node-degree distribution are compared with an exponentially tempered Pareto distribution, $\mathrm{P}\left(X>k\right)\propto {k}^{-\alpha }{e}^{-c\cdot k}$.

**Figure 4:**
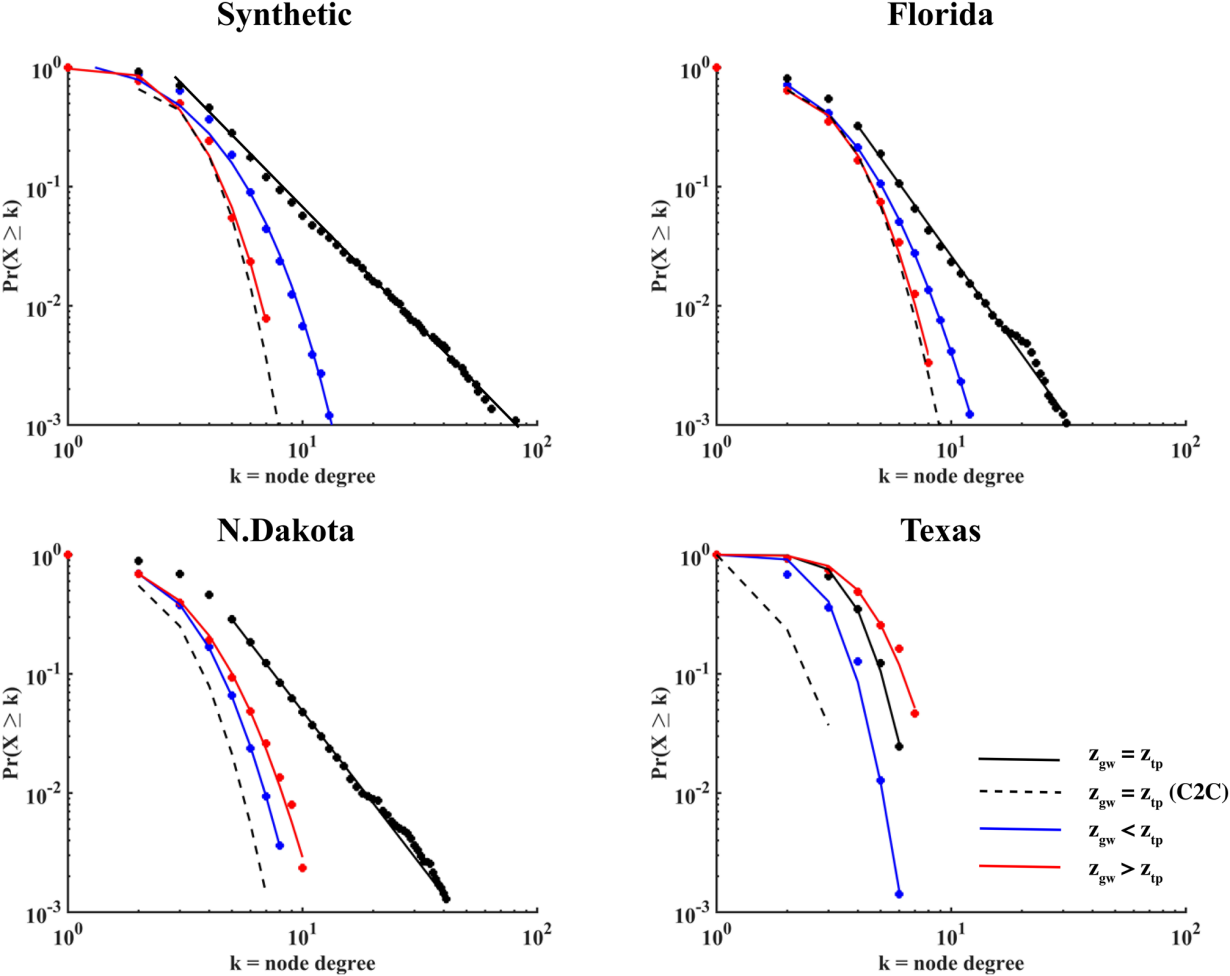
Comparisons between the node degree distributions obtained for C2C (dashed line) and P2P configurations (points), where the latter are shown at different censoring levels, ${\mathrm{z}}_{\mathrm{gw}}$. The solid lines are fits of exponentially tempered Pareto distributions. The node degree distribution for the C2C configuration showed low variability with censoring level, so here only ${\mathrm{z}}_{\mathrm{gw}}={\mathrm{z}}_{\mathrm{tp}}$ is shown; other censoring levels are reported in Supporting Information.

The shape of $\mathrm{P}\left(X>k\right)$ obtained from connecting the wetlands using the P2P criterion varies from an exponentially tempered Pareto distribution ($c>0$) to a Pareto distribution ($c=0$). Except for Texas, $\mathrm{P}\left(X>k\right)$ is Pareto when ${z}_{gw}={z}_{tp}$ in every case for the P2P configuration (Fig. 4), but with different scaling exponents (2.00 $<\alpha <$ 2.75), while all C2C networks are described by similar exponentially tempered $\mathrm{P}\left(X>k\right)$. When ${z}_{gw}={z}_{tp},$ the total perimeter is at maximum, and size distribution heterogeneity is the largest because most wetlands are inundated. At this censoring depth, the majority of wetlands are connected by small P2P gap distances, while few are connected by large P2P gap distances. At other censoring levels (merging or drying wetlands), the parch complexity decreases and the tempering of the Pareto distributions increases (*c* > 0), since the number of wetlands and their sizes decrease, while the mean P2P gap distances increase. On the other hand, the dispersal network for a given wetlandscape constructed using the C2C criterion, is not as sensitive to temporal fluctuations in ${z}_{gw}$ as P2P networks because wetland centroids do not shift significantly during merging-filling-splitting dynamics with (see Supporting Information). The C2C and P2P networks are characterized by the same exponentially tempered Pareto in the Texas wetlandscape because of the relative homogeneity of the landscape, with nearly circular shapes and more regular spatial pattern. The only difference between the C2C and P2P distributions is related to the mean radii of the circular wetlands.

For a given patch habitat landscape, dispersal distance (*D*) of the focal species also affects patch connectivity. As the distance threshold is increased, the Pareto networks are maintained in the P2P configuration, with increased scaling exponent, $\alpha$. Despite the increase in the distance threshold, the node-degree distributions for the C2C configuration still shows an exponentially tempered Pareto $\mathrm{P}\left(X>k\right)$, with an increased mean degree and tempering constant, $c$. However, when the threshold distance, $D$, is much larger than wetland average size, the difference between the C2C and P2P approaches becomes irrelevant and the two node-degree distributions overlap completely.

## Discussion

### Key findings

The overarching goal of this study was to assess how differences in hydrological conditions and landscape complexity affect the structure of ecological dispersal networks in wetlandscapes. The novelty and importance of our work is in coupling spatiotemporal dynamics of landscape complexity to derive a time-varying ecohydrological framework. To further assess how wetland heterogeneity affects variation in network topology, we compared patch connectivity based on the centroid-to-centroid and perimeter-to-perimeter distances.

We found that Florida and North Dakota wetlandscapes and the synthetic fractal landscape have similar features in terms of random spacing of centroids and clustering of P2P gap distances driven by heterogeneity in the distribution of areas and perimeters. Texas wetlands are characterized by low spatial heterogeneity: low abundance, circular shapes and low variance in C2C and P2P gap distances. These differences in spatial heterogeneity are also reflected in the larger variance in connectivity in Florida, N. Dakota and synthetic landscapes, and lower variance in the topology of C2C and P2P networks in the Texas landscape.

The emergence of a Pareto node-degree distribution in $\mathrm{P}\left(X>k\right)$ is related to three key metrics of the wetlandscape derived from DEM data: (1) number of inundated wetlands (i.e., patch density), (2) size and shape distribution (i.e., total wetted perimeter, and fractal dimension), and (3) distribution of gap distances among wetlands. All these features are subject to spatiotemporal dynamics under hydroclimatic forcing, evaluated here using a DEM depth-censoring approach. We showed that for a given wetlandscape, the C2C network serves as the lower bound of all $\mathrm{P}\left(X>k\right)$. When the censoring level is within a critical range for which the abundance of inundated wetlands and their total perimeter are maximized, (1) the spatial distributions of the wetlands show clustering, and (2) the resulting Pareto $\mathrm{P}\left(X>k\right)$ for the P2P network serves as the upper bound for all the node-degree distributions. As landscape heterogeneity and spatial complexity decrease, $\mathrm{P}\left(X>k\right)$ shows increasing tempering of Pareto distributions, typical of random networks, are observed with decreased spatial complexity.

Conceptualizing wetlands as 3-D objects (not points or 2-D shapes) is important for detecting changes in wetland sizes and shapes, and thus shifts in P2P connectivity. The differences we observed in topology of P2P and C2C networks affirms that patch geometry (fractal shape and bathymetry) and size heterogeneity are critical features when computing network topological metrics. For example, using patch centroids may bias the spatial location of nodes when patches tend to be elongated in shape, or inter-patch distances are small relative to the patch dimensions^46^. Temporal dynamics of wetland size, and thus wetland geometrical attributes, are only accounted for in P2P networks. These patterns are also typical of percolation processes unfolding on a fractal surface^47,48^, with thresholds for area and perimeter of wetland patches.

### Ecological implications

The two criteria (C2C; P2P) used to connect wetland nodes significantly affected the network topology. This differentiation is important for a wide range of species that inhabit multiple niches within a wetland. Wetland perimeters are key habitats for amphibians; for example most anurans (e.g., frogs and toads) and many caudates (e.g., salamanders and newts) lay their eggs in shallow water near shorelines, have aquatic larvae, and inhabit forests or other uplands habitats as adults^49^.

For dispersal of birds that feed in open water, the centroids of the wetlands that coincide with open water are more important. The dispersal distance of these birds is much larger than that for amphibians^50^. Similar considerations may be extended also for hydrophytic vegetation associated with wetlands, since certain types of plants prefer habitats along wetland edges^18^, while other emergent or submerged vegetation are found only near wetland centers (e.g., cypress domes). Therefore, the same wetlandscape could sustain diverse dispersal networks based on the preferential wetland zone occupied by a given species and the way they disperse.

Pareto $\mathrm{P}\left(X>k\right)$ networks were found in spatially complex wetlandscapes only for a small range of conditions, suggesting that this configuration is highly sensitive to hydrological variability, either from stochastic hydroclimatic forcing during a year, or during extreme droughts or floods. Land-use change (e.g., groundwater withdrawals) or climate-change (e.g., increasing aridity) also decrease the likelihood of Pareto networks. For example, lowering the groundwater level through pumping wells decreases the number of inundated wetlands, with a reduction in habitat choices and diversity. In addition, P2P gap distances increase under lower water levels, thus changing the accessibility of habitats for a given dispersal distance. Hydrologic thresholds (embedded here in censoring level, ${z}_{gw}$) have important effects on the suitability of wetlands as aquatic habitats, and specific flora and fauna thus need to adapt to cope with time-variable conditions in wetlands^51^.

Differences in C2C and P2P networks are important for the dispersal of species with habitat preferences. For example, “specialist” species could only survive under stable conditions. Our results suggest that such conditions prevail only when the censoring level fluctuates within the narrow range [${z}_{aw};{z}_{tp}$] in which semi-aquatic species have multiple habitat choices to live, breed and disperse. Under these conditions, P2P network topology is characterized by a Pareto $\mathrm{P}\left(X>k\right),\mathrm{ where a}$ small number of large wetlands act as “hubs” in the dispersal network connecting a large number of small wetlands. On the other hand, "generalist" species could readily adapt to a wide range of hydrological conditions^12^, and their dispersal ability could overcome the limitation imposed by the shifts in wetlandscape network topology. Adapting to dynamic habitat conditions with a sub-optimal dispersal network and habitat distribution is essential for co-evolution of resilient aquatic metacommunities^52^.

### Limitations and final remarks

Despite the generality of the DEM-censoring framework proposed here to examine wetlandscapes, it is important to highlight the inherent limitations. For example, our method considers the uplands within which wetland habitats are embedded as “neutral” to species dispersal. Instead, this matrix could be characterized by heterogeneous land cover over which the focal species migrate between patches (nodes). The upland matrix can limit or facilitate species dispersal depending on matrix attributes, such as land cover, migration barriers, or soil–water status^53^. These matrix effects can be accommodated by assigning weights to the network links. Dispersal of individuals is also a function of the biological characteristics and life-history traits of the considered species. For example, during breeding season, adult amphibians migrate from overwintering sites to ponds to mate and deposit eggs^33^. Thus, edges linking patches may be undirected or directed (e.g., different in- and out-degrees) and anisotropic (with preferred directions). The same species could also be characterized by different dispersal strategies based on the age group (e.g., adults vs juveniles), thus, the dispersal distance, $D$, could be weighted based on the life stage of the individual species considered. In addition, dispersal pathways are considered here as Euclidian gap distance, while actual dispersal paths may be more complex.

We start here with the simplest case (no edge weights; undirected; isotropic) to isolate the roles of hydroclimatic forcing and landscape features, but links can be weighted to account for anisotropy and matrix effects. Finally, our DEM-based model is useful to estimate the range of temporal patterns of wetland inundation at the landscape scale, accounting for heterogeneities at local scale. However, when local relief and vertical heterogeneities are too large, or in the presence of river networks or large lakes^54^, or strong soil layering, the assumption of characterizing wetland dynamics using censoring levels parallel to the topography is inadequate, thereby violating the basic assumption of using DEM depth-censoring we used here.

## Conclusions

Hydroclimatic forcing, manifested in shallow groundwater dynamics, is the dominant driver of spatiotemporal fluctuations in the hydrology of wetlandscape habitats, which in turn impacts the dynamics of species dispersal networks. The proposed approach couples the spatiotemporal variability in wetlandscape attributes and connectivity, an essential starting step for modeling metapopulation dynamics in changing patchy habitats.

Our results show that different criteria used to connect wetland nodes in the significantly affect the topology of the resulting dispersal network. When connectivity is based on the perimeter-to-perimeter criteria, and when the groundwater level reaches a critical range for which the number of active wetlands and the total perimeter are maximized, we observe the emergence of Pareto node-degree distributions. Pareto node-degree distributions appears in wetlandscapes for a small range of hydroclimatic conditions suggesting that this optimal configuration is highly sensitive. Therefore, species must adapt and co-evolve with such variable conditions to persist in the wetlandscapes and develop a of resilient aquatic metacommunities.

The C2C and P2P networks may be viewed also as the representation of two different dispersal strategies within the same wetlandscape. In particular, they could represent the two end-members of a spectrum of dispersal strategies of several species that rely on different zones of wetland habitat. Evaluating wetlandscape connectivity is dependent on the type of species of interested, since, for the same wetlandscape, different types of species could have their own connected network. In this perspective, our framework serves as the first step in patchy habitat characterization, fundamental for the development of models for spatial patterns in species occupancy and persistence, which may be used to project the impacts of climate forcing and land cover change on landscape biodiversity in wetlandscapes.

## Supplementary information

Supplementary information
