# Supplementary material for: Emergent dispersal networks in dynamic wetlandscapes
Source: Sci Rep. 2020 Sep 7;10:14696. doi: 10.1038/s41598-020-71739-8 (PMC7477191; doi:10.1038/s41598-020-71739-8)
Supplement: Supplementary file 1 — Supplementary information [file 41598_2020_71739_MOESM1_ESM.docx]

**Supporting Information For**

Emergent Dispersal Networks in Dynamic Wetlandscapes

Leonardo E. Bertassello*^1^, Antoine F. Aubeneau^1^, Gianluca Botter^2^,

James W. Jawitz^3^, and P. S. C. Rao^1,4^

^1^Lyles School of Civil Engineering, Purdue University, West Lafayette, IN 47907-2051, USA

^2^Department of Civil, Architectural and Environmental Engineering,

University of Padua, I-35100 Padua, Italy

^3^Soil and Water Sciences Department, University of Florida, Gainesville, FL 32611, USA

^4^Agronomy Department, Purdue University, West Lafayette, IN 47907-2054, USA

Corresponding author: Leonardo E. Bertassello ([lbertass@purdue.edu)](mailto:lbertass@purdue.edu))

**For Submission to:** *Scientific Reports*

**S1: Wetlandscapes geographical location**

Here, we report the spatial location of the three wetlandscape considered in our analysis. The three maps were obtained using the software QGis 2.14.15 – Essen (https://www.qgis.org/en/site/). The DEM of the regions is reported in the background, while the red polygons represent the wetland mapped by the National Wetland Inventory databased (https://www.fws.gov/wetlands/).


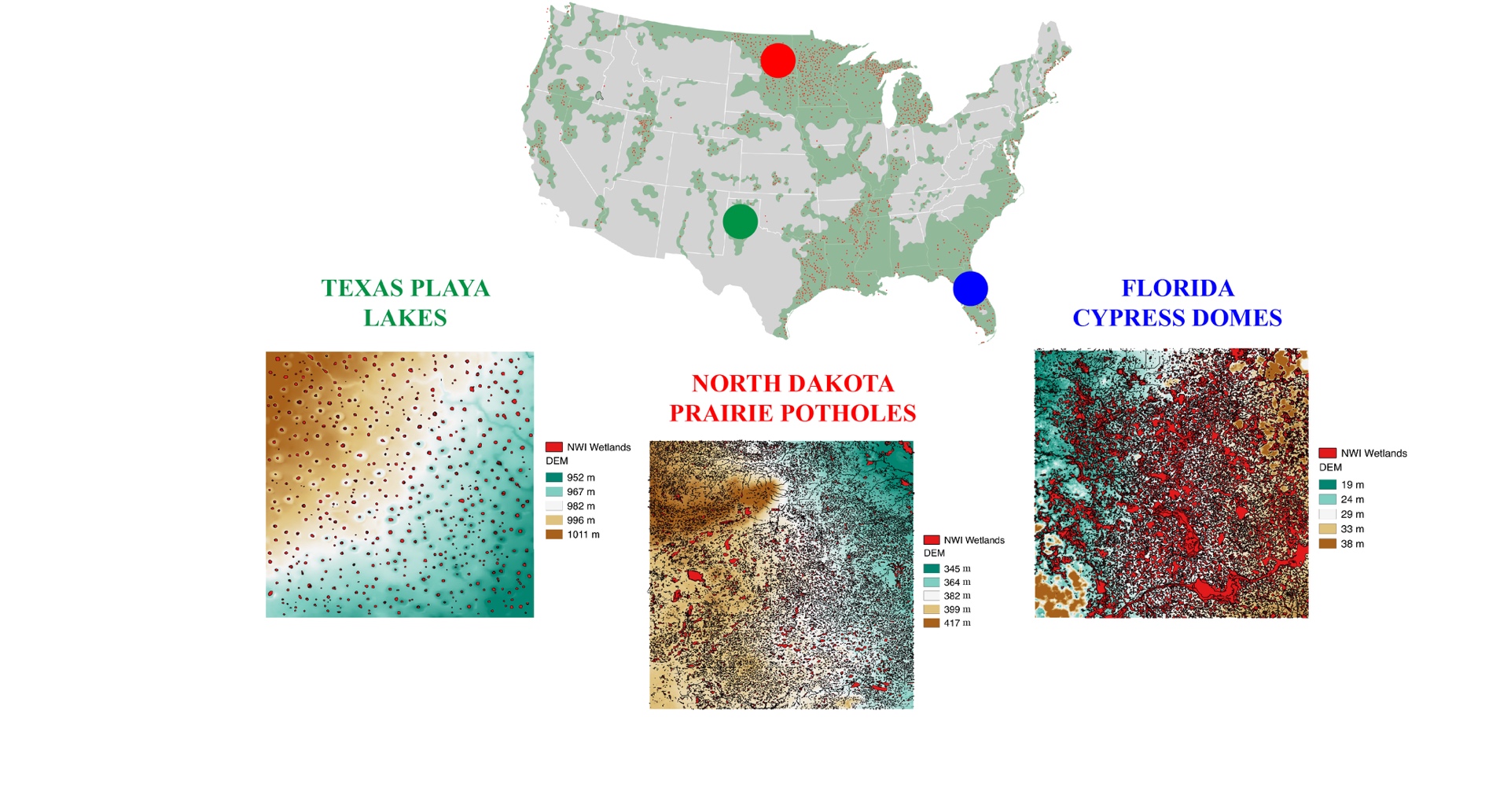


Figure S1: Geographic location of the three U.S. wetlandscapes (30 x 30 km) used as case studies in present analyses. The three DEMs of the region is reported in the background. Furthermore, we added the wetlands listed in the National Wetland Inventory database as reference (red polygons).

**S2: Heterogeneity in DEMs**

We assessed the degree of heterogeneity of landscape topography in the four DEMs by computing the Hurst exponent, H, for different moving windows of the spatial domain. For each moving square moving window of size 1 km x 1 km we estimated the Hurst exponent. The value of H is calculated using well-accepted variogram method (Voss, 1988; Gallant et al., 1994). The semi-variogram, $\gamma\left( l \right)$, is given by:

$$\gamma\left( l \right)=\frac{1}{2N(l)}\sum_{i=1}^{N(l)} \left[ Z\left( s_{i}+l \right)-Z(s_{i}) \right]^{2} (S1)$$

where, $s_{i}$ is location $i$, $l$ is the lag distance, $Z(s_{i})$ is the elevation at location $s_{i}$, $Z\left( s_{i}+l \right)$ is the elevation at location ($s_{i}+l$), and $N(l)$ is the number of pairs spaced at $l$. Fractional Brownian motion (fBm) corresponds to a particular form of the semi-variogram, $\gamma\left( l \right)\propto l^{2H}$. Thus, the slope $S$ of the best-fitting line produced when the logarithm of the distance between the sampled points is regressed against the logarithm of the mean-squared difference in the elevation for that distance is related to the Hurst exponent by the expression: $H=S/2$ (Russ, 1994),

Figure S2: Spatial variation of the Hurst exponent, H, in the four analyzed DEMs.

**S3: CSR test for multiple censoring levels**

We tested the randomness in the distribution of wetlands conceived both as points (C2C) and 2D objects (P2P) for different censoring levels, $z_{gw}$. Figure S3 shows the results of the CSR test for the synthetic generated landscape at four $z_{gw}$ values. When the censoring level is low, the difference between the C2C and P2P nearest neighbor difference is limited and they both follow a spatial random distribution. Instead, as the censoring level increases and thus more wetlands fill and their size gets bigger, we observe discrepancy between conceiving wetlands as points vs two-dimensional objects. In particular, the CSR is still satisfied for the C2C configuration, but not for the P2P.

Figure S3: Comparison of CSR test for different censoring level in the synthetic generated landscape for the P2P and C2C nearest neighbor distances. When the data follow the 1:1 line, the spatial point pattern is described by a CSR process.

**S4: Sensitivity of node-degree distribution on threshold distance, D**

We assessed the sensitivity of the node-degree distribution on the distance threshold, $D$, in the four case studies, both for the C2C and P2P configurations. All the following results are obtained when $z_{gw}=z_{tp}$. With the exception of Texas, for all the other analyzed cases, the P2P configuration allow the emergence of scale free distributions (tempering constant, c = 0), while in all the C2C networks, the node degree distribution is exponentially tempered. As the distance threshold, $D$, is increased, the Pareto networks are maintained in the P2P configuration, with increased scaling exponent, $\alpha$

**** Figure S4: Comparison between the node-degree distribution in the C2C and P2P configurations for D = 100 m in the Synthetic-generated landscape, Florida and N. Dakota, and D = 500 m in Texas.

Figure S5: Comparison between the node-degree distribution in the C2C and P2P configurations for D = 200 m in the Synthetic-generated landscape, Florida and N. Dakota, and D = 1000 m in Texas.

Figure S6: Comparison between the node-degree distribution in the C2C and P2P configurations for D = 500 m in the Synthetic-generated landscape, Florida and N. Dakota, and D = 1500 m in Texas.

**S5: Correlation between number of wetlands and total perimeter with Hurst exponent**

Figure S7: Correlation between number of wetlands and total perimeter with Hurst exponent in the four case studies. The exponent of the power law is obtained from fitting the rising limb of the curves presented in Figure 2 with the function: $y\propto x^{\beta}$.
